# Long non-coding SINEUP RNA enhancing BDNF translation prevents dendritic atrophy following an Aβ-peptide challenge

**DOI:** 10.1007/s00018-026-06175-4

**Published:** 2026-03-14

**Authors:** Marta Atzori, Elsa Fabbretti, Viviana Ciraci, Silvia Zucchelli, Carlotta Bon, Sanja Mikašinović, Gabriele Baj, Stefano Gustincich, Enrico Tongiorgi

**Affiliations:** 1 Department of Life Sciences, University of Trieste, Via Licio Giorgieri, 5 (Q building), Trieste, 34127 Italy; 2 Center for Human Technologies, Non-coding RNAs and RNA-based therapeutics, Italian Institute of Technology (IIT), Genoa, 16152 Italy

**Keywords:** Alzheimer’s diseases, Neuronal atrophy, Dendritic dystrophy, Neuroprotection, Long non-coding RNA, Gene-therapy

## Abstract

In Alzheimer’s disease (AD), brain levels of Brain-derived neurotrophic factor (BDNF) are reduced. BDNF, a neurotrophin involved in neuronal development and plasticity, has a protective role against the cytotoxic effects of Aβ, a pathological hallmark of AD. Since therapeutic approaches to increase BDNF levels by supplementing BDNF directly in the nervous system have been ineffective, we explored an alternative strategy: to increase intracellular BDNF levels by using synthetic SINEUP long non-coding RNAs which upregulate protein synthesis without altering mRNA levels. We designed one SINEUP common to all BDNF transcripts (SINEUP-PAN) and one SINEUP complementary to the BDNF 5’exon-I transcript (SINEUP-Ex1), which is downregulated in AD and not expressed in non-neuronal tissues. SINEUPs activity and specificity have been first proven on endogenous targets in different mammalian cell lines – human neuroblastoma cells, rat neurons and mouse astrocytes. During in vitro development of naïve primary rat hippocampal neurons, SINEUP-Ex1, but not SINEUP-PAN, induced more complex dendritic arborisations at 12 days in vitro (DIV12). In DIV6 neurons, 24 h incubation with aggregated Aβ_25−35_ peptide caused neuronal atrophy and both SINEUP-PAN and SINEUP-Ex1 promoted the full rescue of soma size, apical dendrite diameter, and number of dendritic endpoints, but not of the total dendritic length. The protection from the Aβ-challenge achieved with the two SINEUPs appear to involve distinct mechanisms, as only SINEUP-PAN was able to increase Map2, a marker of dendritic microtubules which show aberrations in AD. These results support SINEUP as a potential new tool for a gene-therapy in AD and other neurodegenerative diseases.

**Supplementary Information:**

The online version contains supplementary material available at 10.1007/s00018-026-06175-4.

## Introduction

Dendritic atrophy in the absence of neuronal cell death is a common finding in the early phases of Alzheimer’s disease (AD), when only mild cognitive impairment symptoms are detected [1–4]. AD is characterized by two pathological hallmarks, amyloid-β (Aβ) plaques and tau aggregates. During initial AD phases, soluble forms of oligomeric Aβ peptides are produced, causing a loss of synaptic plasticity associated with a triad of morphological changes including dendrite simplification, spine loss and neurites dystrophies [2]. Several studies observed an enhanced vulnerability of neurons to Aβ peptides injuries as a result of age-dependent lowering of brain levels of neurotrophic factors [5–8]. Brain-derived neurotrophic factor (BDNF) is one of the main factors whose expression is down-regulated in the brains of AD animal models and patients, contributing to reduced trophic support of neurons [9–12]. Notably, reduced BDNF levels are associated with dendritic atrophy and neuronal soma shrinkage, possibly through a dysregulation of cytoskeleton dynamics [13]. In addition, a decrease in BDNF levels causes a drop in 5’CAP-dependent protein translation through the mTOR pathway, leading to reduced synaptic protein levels and defective synaptic function and morphology [14]. Altogether, these studies suggest that restoration of physiological BDNF levels may have strong therapeutic potential for a protective treatment in AD [15–18]. However, delivery of correct amounts of BDNF into the appropriate brain locations represents a major technical challenge.

To reach optimal effects, BDNF levels have to be maintained within restricted limits (both upper and lower) of physiological concentrations in order to avoid side-effects like epilepsy (too high doses) or lack of efficacy (too low doses). However, attempts to deliver BDNF into the brain through systemic supply have failed and small BDNF analogues have shown, to date, limited efficacy [19, 20]. Various drug screening assays were designed to identify compounds able to increase BDNF mRNA [21], but mRNA levels are only an indirect measure of protein levels. In fact, BDNF protein expression levels are the result of translation of multiple transcripts generated by 8 exons encoding the 5’UTR sequences that are alternatively spliced to a common downstream exon (exon IX) containing the coding sequence (CDS) and the 3’UTR region with two polyadenylation sites. The resulting 22 mouse/rat (36 in humans) BDNF transcripts have distinct patterns of expression [22–24] and produce different amounts of BDNF protein basally, and in response to various stimuli [25]. Furthermore, it has recently been shown that ablation of individual transcripts encoding exons I, II, IV or VI in mice is associated with different behavioural deficits [26]. The BDNF transcript containing the exon I is an interesting target for a gene therapy because it is downregulated in AD and is highly expressed in the brain but is virtually absent in other non-neuronal tissues [22, 23].

In contrast to all previous approaches, we propose an innovative strategy to enhance BDNF levels through a non-coding RNA-based therapy in which enhancement of the translation of individual endogenous BDNF transcripts is achieved. The SINEUPs are a new functional class of natural and synthetic antisense (AS) long non-coding RNAs (lncRNAs) that activate translation and represent an original RNA-based therapy [27]. The name SINEUP derives from both their structure and their function: “SINE” refers to the inverted SINEB2 sequence by which the lncRNA is composed, while “UP” underlines its ability to upregulate translation. A SINEUP is typically composed by two RNA domains: the Binding domain (BD), able to recognize and overlap with the target mRNA sequence, and the Effector domain (ED) or SINEB2 sequence, essential to enhance translation [27–29]. Recently, EDs have been shown to function in SINEUPs as Internal Ribosomal Entry Site (IRES) in *trans*, guided by an antisense sequence. This conclusion stems from the observation that the inverted SINEB2 sequence displays IRES activity in *cis* and that both viral and cellular IRES elements can function as EDs in synthetic SINEUPs [30].

The representative member of SINEUP is the AS Uchl1, which increases the translation of UCHL1 (Ubiquitin carboxyterminal hydrolase L1) whose protein expression is downregulated in familial Parkinson’s disease and in other neurodegenerative diseases [31, 32]. Synthetic SINEUPs can be engineered to enhance the translation of specific transcripts with therapeutic potential. Thanks to the versatility of BD design, mRNAs with distinct 5′UTR sequences can be selectively targeted. Importantly, SINEUPs increase endogenous protein levels only when the target mRNA is expressed and by approximately 1.5- to 2.5-fold, avoiding the toxicity associated with ectopic and large overexpression. A synthetic SINEUP non-coding RNA was shown to rescue defective haploinsufficient gene dosage in a medakafish model of microphthalmia with linear skin defects (MLS) syndrome, a X-linked dominant human disorder, leading to amelioration of the disease phenotype [33]. In patient-derived cellular model systems, SINEUP RNA was able to restore physiological expression of frataxin and mitochondrial activity in Friedreich’s Ataxia, molecular phenotypes associated with CHD8 suppression in autism spectrum disorder and UBA5 expresssion in encephalopathy [34–36]. A SINEUP targeting glial-derived neurotrophic factor (GDNF) was also able to rescue motor deficits and neurodegeneration in a mouse model of Parkinson’s disease [37]. In this study, we explore the effects of two SINEUPs with distinct BDs on endogenous BDNF in primary rat hippocampal neurons. We investigated the total dendritic length (TDL) and other neuronal morphogenesis features after BDNF-targeted SINEUPs expression in hippocampal neurons using an automated image analysis. Finally, we investigate their effect in hippocampal neurons under stress conditions induced by a 24 h Aβ_25−35_ treatment.

## Materials and methods

### DNA plasmids and SINEUP

To express different 5’UTR-BDNF rat transcripts, the following plasmids were used: pEx1-BDNF-GFP (transcript variant 2, NM_012513.4), pEx2B-BDNF-GFP (transcript variant 4, NM_001270632.1), and pEx2C-BDNF-GFP (transcript variant 3, NM_001270631.1). All BDNF mRNA constructs were cloned under a CMV promoter [38]. The pEGFP-N1 plasmid (Clontech) was used as a negative control. Each miniSINEUP construct is transcribed under a CMV promoter.

miniSINEUP-PAN (SINEUP-PAN) was designed to target a sequence common to all rat BDNF transcript variants. The binding domain (BD) spans nucleotides − 22 to + 4 relative to the initiating M1-AUG codon (5’-TCATCACTCTTCTCACCTGGTGGAAC-3’). miniSINEUP-Ex1 (SINEUP-Ex1) was designed to specifically target rat BDNF transcript variant 2 *(*NM_012513.4*).* Its BD spans nucleotides − 40 to + 4 relative to M1 (5’-ACATTGTGGCTTTGCTGTCCTGGAGACTCAGTGTCTTAAAATCT-3’).

All binding domains exhibit 100% sequence homology with the corresponding mouse BDNF transcripts, ensuring cross-species functional conservation. Notably, SINEUP-Ex1 targets mouse BDNF transcript variant 1 *(*NM_007540.4*).* Additionally, a shorter binding domain, common to all rat and mouse transcript variants, was identified and synthesized in SINEUP-PAN2. This minimal BD spans nucleotides − 14 to + 4 relative to the M1-AUG (5’-TCATCACTCTTCTCACCT-3’), and may be used for universal targeting across isoforms.

The use of SINEUPs to increase BDNF expression for any commercial and therapeutic purposes, including BDNF industrial protein production and gene-therapy use is covered by the patent ITALIA N. 102,023,000,026,952 with an international extension PCT/IB2024/062610, owned by the University of Trieste and the Italian Institute of Technology.

## Cell cultures and transfections

HEK293T cells were routinely cultured in DMEM supplemented with 10% of fetal bovine serum and 1% penicillin-streptomycin (Euroclone). For transfection, 3 × 10^5^ cells/ml HEK293T cells were seeded and used after 24 h. Transfection of different plasmids in the appropriate ratio was done using TransIT-X2 Dynamic Delivey System (Mirus) following manufacturer’s instructions. For each condition, 130 ng of pEX1-BDNF-GFP plasmid DNA was used, and co-transfected with increasing concentrations of plasmids encoding the SINEUPs at the ratio 1:1 (130:130 ng), 1:3 (130:390 ng), 1:4 (130:520 ng), 1:6 (130:780 ng). Transfection efficiency was 70–80%. C8-D1A astrocytes were routinely maintained in DMEM supplemented with 10% fetal bovine serum and 1% penicillin-streptomycin (Euroclone). For transfection, 3 × 10⁵ cells per well were seeded in 6-well plates and allowed to adhere for 24 h. Transfections were performed using Lipofectamine 2000 (Invitrogen), according to the manufacturer’s instructions.

Hippocampal neurons were prepared from postnatal day 0–1 from Wistar rats, as previously described (Baj et al., 2016). Animals were treated according to the institutional guidelines, in compliance with the European Community Council Directive 2010/63/UE for care and use of experimental animals. Authorization for animal experimentation was obtained from the local ethical committee on November 10th 2017 and was communicated to the Italian Ministry of Health, in compliance with the Italian law D. Lgs.116/92 and the L. 96/2013, art. 13. All efforts were made in order to minimize animal suffering and to reduce the number of animals used. The hippocampi were collected in 1.8 mL of cold Hank’s balanced salts solution containing: NaHCO3 4.2 mM, Hank’s salt powder 0.952%, HEPES (4-(2-hydroxyethyl-1-piperazineethanesulfonic acid) 12 mM (Sigma), D-glucose 33 mM, kynurenic acid 100 mM, penicillin-streptomycin 1 mg/ml (Euroclone). Hippocampi were digested with Trypsin (0.25%) for 8 min at 37 °C. Cells were seeded at a concentration of 240000 cells/ml (640 cell/mm^2^) on poly-L-ornithine pre-coated plates in presence of a layer of 2% Matrigel (Corning). Cells were grown in Neurobasal medium supplemented with B27 (Life Technologies), 1 mM L-glutamine and antibiotics (Euroclone). At days in vitro 3 (DIV3), the culture medium was changed with fresh one supplemented with 2.5 mM cytosine b-D-arabinofuranoside (Ara-C). Transfection of plasmids in primary hippocampal neurons was performed in cultures at DIV3 using Lipofectamine 2000TM (Thermo Scientific) following manufacturer’s instructions. For each condition, a maximum of 1 µg of total plasmid DNA was used. The transfection efficiency in neurons was 14%.

SH-SY5Y cells were cultured with complete DMEM/F12 (supplemented with 10% of fetal bovine serum (FBS) and 1% of Penicillin/streptomycin) in a humidified, 5% CO_2_, 37 °C incubator. Cells were cultured without coating. The SINEUPs and GFP plasmid were transfected with Lipofectamine 2000 according to the manufacturer’s instructions. In brief, one day before transfection 3,5 × 10^5^ cells were seeded in 2 ml of growth medium on 6-well plates. On the day 1, SINEUPs (1 µg) and GFP plasmid (1 µg) were transfected with lipofectamine 2000 (4 µl) into cell for 4 h, after medium was changed. The cells were incubated at 37 °C in a CO2 incubator for 24 h and then stopped with lysis buffer to extract proteins.

## Aβ treatments

For Aβ treatments, the Aβ_25−35_ peptide (Bachem, Swiss) stock solution was prepared dissolving the peptide in sterile water at a concentration of 5 mg/ml (Copani et al., 1995). Aβ_25−35_ aggregation was obtained in phosphate buffer solution (PBS) for 24 h at 37 °C [39]. The aggregated Aβ_25−35_ oligomers were stocked at 4 °C and used within few weeks from the preparation. Primary cell cultures of hippocampal neurons previously transfected with SINEUPs plasmids at DIV3 were treated with Aβ_25−35_ (10 µM) in culture medium at DIV5 for 24 h to evaluate the effect of Aβ_25−35_ at DIV6.

## Total protein extracts and western blot

Hippocampal neurons lysates were prepared 24 h from transfection, in ice-cold RIPA lysis buffer (NaCl 150 mM, Triton X-100 1%, sodium deoxycholate 0.5%, EDTA 1mM, Tris 50 mM, pH 8) supplemented with a protease inhibitors cocktail (Roche). Bradford assay (Abcam) was used to determine protein concentration and ensure correct loading on SDS-PAGE. For Western blot, membranes were incubated with the mouse monoclonal anti-BDNF recognizing both BDNF forms (Sigma Cat. #B5050, dilution 1: 1000, over-night at 4 °C). and with HRP-conjugated secondary antibody (Sigma, dilution 1:20000;). Signals were obtained with ECL Prime Western blotting detection reagent (GE Healthcare Life Sciences). For gel loading controls, membranes were stripped for 30 min in Restore PLUS WB stripping Buffer (Thermo Fisher), washed and incubated with anti-αTubulin antibody (GeneTex, dilution 1:10000). Signal intensity was then quantified with ImageJ.

HEK293T cells and SH-SY5Y neuroblastoma lysates were prepared from 3,5 × 10^5^ cells/wells seeded onto 6-well plate. For each well 70 µl of ice-cold RIPA lysis buffer supplemented with HALT protease and HALT phosphatase inhibitors were added. Then, protein extracts were sonicated and centrifuged. 50 µg of protein extracts were separated in 15% SDS-PAGE gel and then transferred onto a PVDF membrane. The membrane was blocked by 5% non-fat milk in TBST- solution (1 h, RT) and then, the HEK293T cells were incubated with the rabbit polyclonal anti proBDNF antibody (Alomone, Cat. # ANT-006, dilution 1:1000, over-night at 4 °C) while the SH-SY5Y cells were incubated also with the mouse monoclonal anti-BDNF recognizing both BDNF forms (Sigma Cat. #B5050, dilution 1:1000, over-night at 4 °C). and mouse anti alpha tubulin (Sigma, Cat. # T6074, dilution 1:3000) over-night at 4 °C. As a secondary antibody were used anti-rabbit 488 (Abcam Olc, 1:1000) and anti-mouse 488 (Abcam Olc, 1:1000). Bands were detected on Chemidoc and quantified with ImageLAB software.

C8-D1A astrocytes lysates were prepared from 3,5 × 10^5^ cells/wells seeded onto 6-well plate. Cells were washed twice with ice-cold PBS 1X, collected with 1 mL of PBS 1X, and splitted into two half for both protein and RNA extraction. Cell pellets were collected by centrifugation at ~ 2500 × g for 5 minutes at 4°C. Proteins were estracted using 100 µl of ice-cold RIPA lysis buffer supplemented with HALT protease and HALT phosphatase inhibitors were added. Mixtures were gently shaken on ice for 15 minutes, then centrifuged at ~ 14,000 × g for 15 minutes at 4°C to pellet cell debris; the supernatants were subsequently transferred to new tubes for further analysis. 20 µg of protein extracts were separated in 4–20% SDS-PAGE. Protein transfer was carried out by using Trans-Blot SD unit (Biorad) with the following condition: semi-dry transfer, 25V 10’, 0.2 μm Nitrocellulose membrane. The membranes were blocked by 5% non-fat milk in TBST- solution (1 h, RT) and then incubated with the mouse monoclonal anti-BDNF recognizing both BDNF forms (Sigma Cat. #B5050, dilution 1: 1000, over-night at 4 °C). As a secondary antibody were used anti-rabbit (Sigma-Aldrich, Cat. #A0545, 1:10000, 1 h at room temperature). As loading control, membranes were incubated with mouse monoclonal peroxidase conjugate anti beta actin (Sigma-Aldrich, Cat. #A3854, dilution 1:20000, 1 h at room temperature). Bands were detected on Chemidoc (BioRad) and quantified with ImageJ software.

## Immunofluorescence microscopy

Hippocampal neurons were fixed in a 4% paraformaldehyde/phosphate buffer (PFA) solution for 20 min at room temperature and incubated with anti-Map2 (GeneTex) and anti-NeuN (Millipore) antibodies (1:500) or the monoclonal anti-BDNF (Cat. #B5050, Sigma at 20 µg/ml, according to [40]. Signals was revealed with anti-IgG Alexa Fluor 568 or Alexa Fluor 488 antibodies (Thermo Scientific) (1:1000 dilution). Nuclei were counterstained using Hoechst dye 33342 (0.001%, Thermo Scientific). Samples were covered with an anti-bleaching reagent (Southern Biotech) to preserve the fluorescence and visualized using Nikon ECLIPSE Ti-E live-imaging epifluorescence microscope, using a 20X objective. Fields were captured using the Nikon acquisition software NIS-elements. NeuN and Hoechst positive cells were counted using the “Object Analyzer” NIS-Elements option. Images were taken with a comparable number of neurons per field. Total dendritic length (TDL) and number of dendrite endpoints was analysed by means of Map2 tracing and quantified with Neurite Quant, an open source plugin for ImageJ [41]. Three cultures were analyzed and for each culture, four images were acquired with 20x in the central region of each well and combined into a single large image by the function “Stitching” of the Nis-Elements software. In microscopy experiments, Map2 signal was quantified to define the area with the highest Map2 fluorescence intensity, setting up an arbitrary fluorescence intensity threshold mask in NIS-Elements.

### Statistical analysis

Data were analysed in Microsoft Excel (Office) and GraphPad Prism 7. Based on Shapiro-Wilk normality test, statistical significance between 2 groups/conditions was obtained by Student-t test in case of positive normality test. For two groups/condition with negative outcome of normality test, Mann-Whitney Rank Sum test was performed to compare two groups/conditions. For multiple comparisons between more than 2 groups/conditions, One-Way ANOVA was performed in case of positive normality test; for not normal data, Kruskal-Wallis test was used.

## Results

### SINEUPs upregulate Ex1-BDNF mRNA translation

**Fig. 1:**
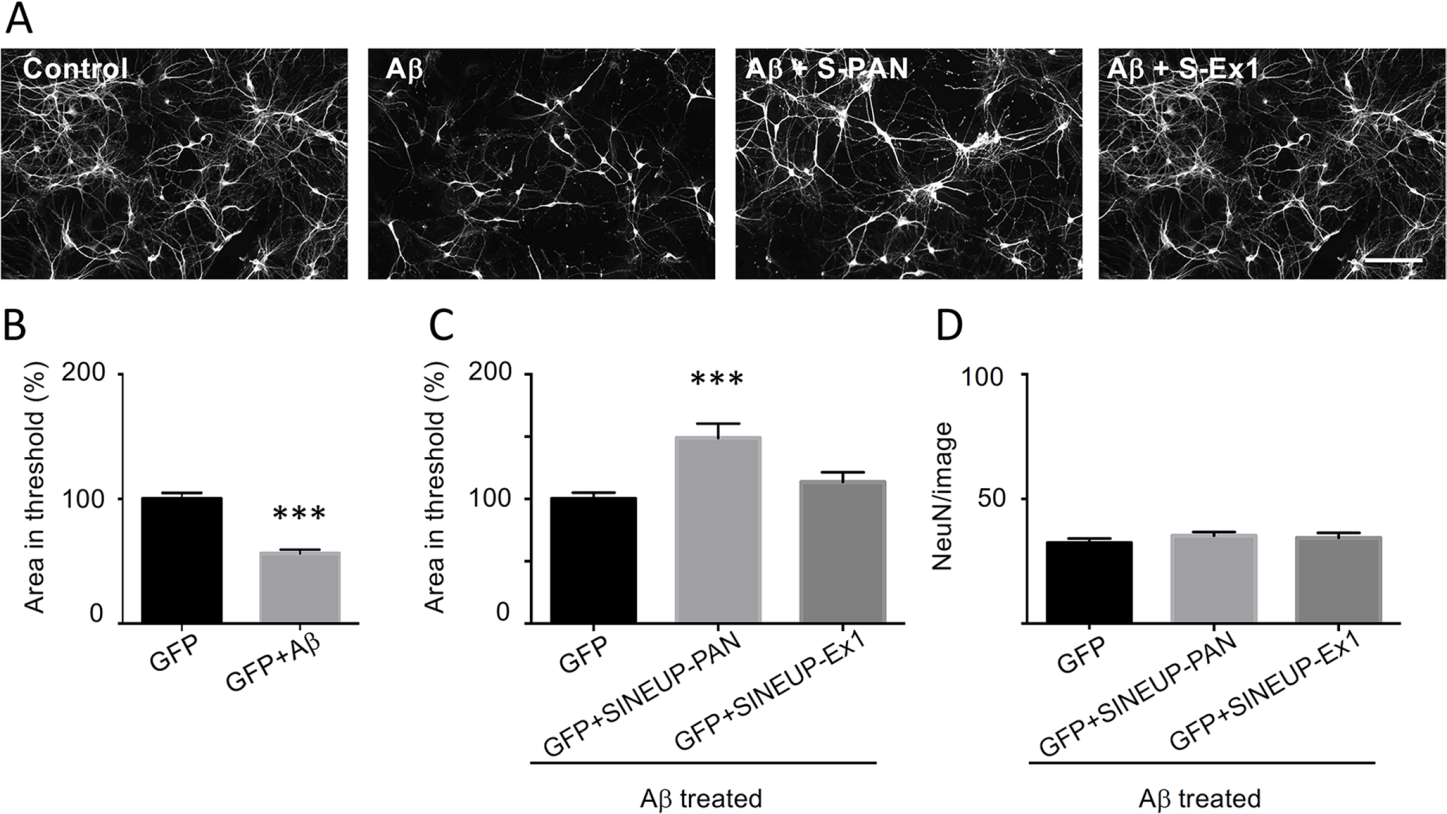
SINEUP strategy, specificity and efficacy in targeting BDNF protein upregulation. **A-B**. Rat BDNF gene structure with schematic representation of BDNF transcripts characterised by multiple alternative splicing of 5’ and 3’UTRs (grey boxes), a coding sequence (CDS, white boxes) and two poly-adenylation sites (polyA). B. SINEUP-PAN and SINEUP-Ex1 annealing sites on BDNF transcripts used in this study. C-H. HEK293T cells are co-transfected with different plasmids to demonstrate the efficacy of SINEUP-PAN or SINEUP-Ex1 on the indicated BDNF transcripts with respect to control (GFP). Plasmids were transfected in 1:3 ratio. Tubulin (55 kDa) is indicative of gel loading and is used for normalization in the Western blot analysis. Histograms quantify average data from *n* = 4 experiments. The molecular weight for the BDNF-GFP band of 64–65 KDa correspond to the predicted size for a chimeric protein made of the proBDNF form (32–34 KDa) and the GFP (27–30 KDa). **C**. Effect of SINEUP-PAN on protein levels encoded by the transfected chimeric Ex1-BDNF-GFP transcript with respect to control represented by the plasmid encoding the reporter protein GFP alone. *p* = 0.0005. **D**. Effect of SINEUP-Ex1 on protein levels encoded by the transfected chimeric Ex1-BDNF-GFP transcript. *p* = 0.0299. **E-F**. Effect of SINEUP-PAN on protein levels encoded by the transfected chimeric Ex2B-BDNF-GFP (E) and chimeric Ex2C-BDNF-GFP (F) transcripts. *n* = 3. **G-H**. Effect of SINEUP-Ex1 on chimeric Ex2B-BDNF-GFP (G) and chimeric Ex2C-BDNF-GFP (H) transcripts with respect to control (GFP). All the quantitative results shown are produced from a repetition of *n* = 3 experiments and the Western blot images show the greatest effect obtained and therefore, are not representative of the average results

BDNF transcripts have a bipartite structure constituted by one out of eight alternative spliced upstream exons, each encoding a different 5’UTR, and one downstream exon identical for all transcripts, bearing the CDS and the 3’UTR (Fig. 1A) [22]. The mechanism of action of SINEUPs involves the formation of an RNA duplex between the SINEUP and its target mRNA at the translation initiation site (AUG). This interaction promotes the recruitment of the translation initiation complex, driven by the IRES activity of the ED, ultimately leading to increased protein synthesis [29, 30].

BDNF-specific SINEUPs were designed to bind a specific target sequence on BDNF transcripts to promote the BDNF protein expression. The SINEUP-PAN targets a sequence located in exon IX at the beginning of the CDS, common to all transcripts, overlapping to most of BDNF transcripts, while SINEUP-Ex1 was designed to specifically recognize BDNF exon 1 (Fig. 1B).

According to the general SINEUP guidelines [32], the amount of transfected SINEUP RNA should be optimized in each experimental condition. Therefore, we run a series of co-transfection experiments in HEK293T cells to define the most appropriate plasmid ratio (Suppl. Figure 1). The plasmids encoding the SINEUP-PAN or the SINEUP-Ex1 were co-transfected with either the pN1-EGFP plasmid encoding the green fluorescent protein (GFP), which represented the control, or with plasmids encoding either the plasmid Ex-1-BDNF containing the exon 1 sequence followed by the BDNF CDS in fusion with the GFP or the pEx2B- or the pEx2C-BDNF-GFP plasmids encoding the exon 2B or 2 C sequences, respectively. The rationale for using exogenous transcripts is twofold. Firstly, to test the efficacy of the SINEUPs by measuring the signal of the reporter gene GFP fluorescence, which is easy to detect and provides an almost linear correlation of the fluoresecence intensity with the protein levels. Secondly, by transfecting single BDNF exons that were traceable by the report gene, it is possible to separate the effect of the SINEUPs on each individual exon. This would have not been possible to achieve by simply targeting the endogenous, untagged, mRNAs, because the natural protein product is identical for all BDNF transcripts. The Supplementary Fig. 1A shows this principle and the specificy of the anti-proBDNF antibody used.

Western blot analysis for BDNF showed that the 1:3 ratio of plasmids SINEUP-PAN/BDNF Ex-1-CDS-GFP induced a significant increase in BDNF protein levels (+ 414.9 ± 110.2%; *p* = 0.049; Suppl. Figure 1B) with respect to control (100 ± 26.49%) and similar results were obtained with the ratio 1:4 and 1:6. The increase was evident also at 1:2, but without reaching the statistical significance. In contrast, at 1:1 ratio there was no appreciable increase in BDNF levels (Suppl. Figure 1B). The results with SINEUP-Ex1 were very similar to those observed with SINEUP-PAN, in that the 1:1 ratio did not produce any increment in BDNF translation, while there was a significant increase in BDNF levels for the 1:2 ratio (+ 271 ± 40.3%, *p* = 0.015) and 1:3 ratio (+ 276 ± 54.57%, *p* = 0.034) and similar increases were present also at 1:4 and 1:6, although with more experimental variability (Suppl. Figure 1 C). In conclusion, the 1:3 appears to be the lowest ratio to achieve the maximal effect on BDNF translation levels and therefore it was used in all subsequent experiments.

In order to verify the specificity and efficacy of SINEUP-PAN and SINEUP-Ex1 on the different BDNF target transcripts, we performed co-transfection experiments of SINEUPs and BDNF plasmids encoding different 5’UTR sequences. The pEx1-BDNF-GFP (Fig. 1C-D) and pEx2B- or the pEx2C-BDNF-GFP plasmids (Fig. 1E-H) were co-transfected in HEK293T cells at the 1:3 ratio with SINEUP-PAN, or SINEUP-Ex1 or pEGFP-N1 alone (GFP) and BDNF protein expression was measured with Western blot. Both SINEUP-PAN and SINEUP-Ex1 significantly upregulated the expression of BDNF protein with respect to control samples, when pEx1-BDNF-GFP was co-transfected with SINEUPs (*p* = 0.0005 and *p* = 0.03; Fig. 1C, D). In addition, SINEUP-PAN upregulated BDNF also in samples transfected with pEx2B-BDNF-GFP or pEx2C-BDNF-GFP, although not at significant level (Fig. 1E, F), while, as expected, SINEUP-Ex1 was not effective on these BDNF transcripts (Fig. 1G, H).

**Fig. 2:**
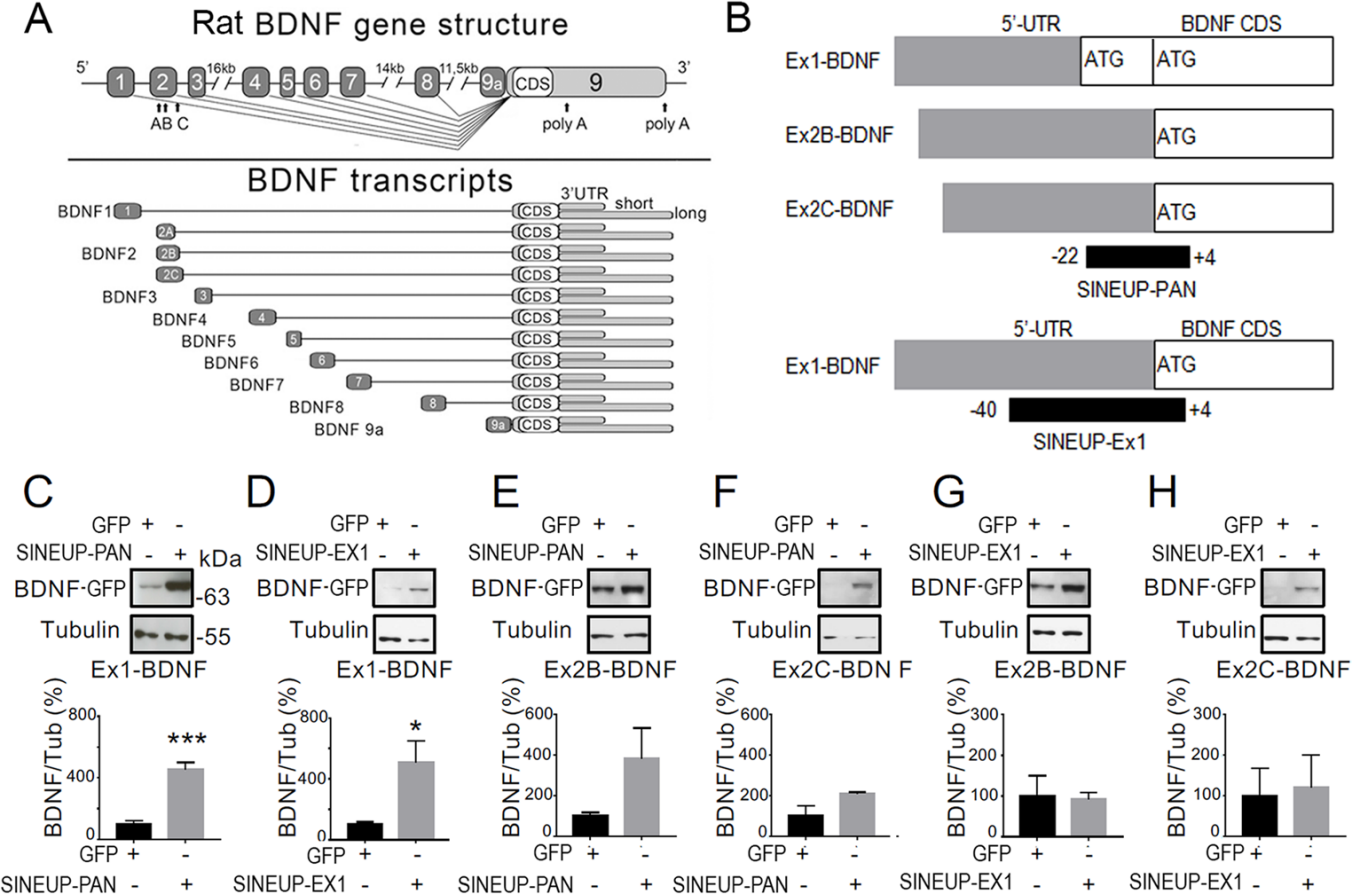
SINEUP efficacy in upregulating endogeous BDNF protein expression. (**A**) Hippocampal neurons transfected with GFP alone (pEGFP-N1), or cotransfected with GFP 1:3 and SINEUP-PAN, or SINEUP-Ex1 at DIV3, and stained at DIV6 by immunoflurescence with anti-BDNF antibody (Ab, αBDNF - Sigma, secondary Ab = Alexa568). Calibration bar = 25 μm. (**B**) Example of a GFP-tranfected neuron on which three regions of interest (ROIs) were designed on the soma (1), the apical dendrite (2) or on a small extracellular region to determine the background level (3). (**C**) Quantification of anti-BDNF immunofluorescence intensity recorded on *N* = 12 neurons for each condition. Histograms show the effect of SINEUP-PAN, or SINEUP-Ex1 on endogenous BDNF with respect to control (GFP). (**D**) Qualitative and quantitative Western blot analysis of endogenous pro-BDNF level in human SH-SY5Y neuroblastoma cells. The SINEUP-PAN significantly increased the level of pro-BDNF (anti-BDNF Alomone, *p* < 0,05; *n* = 4) with respect to GFP (control), while SINEUP-Ex1 did not induce significant changes. (**E**) Qualitative and quantitative Western blot analysis of endogenous mature BDNF level in C8-D1A cells, a mouse astrocytic cell line. SINEUP-PAN, SINEUP-PAN2 and SINEUP-Ex1 significantly increased the level of mature BDNF (anti-mBDNF, Sigma). SINEUP-PAN2 indicates an RNA molecule with the same BD as SINEUP-PAN but shortened to -14/+4. (*n* = 4 or 6 independent cultures). Statistical analysis is one-Way ANOVA with multiple correction (Dunnett) = *p* < 0.01; = *p* < 0.001

In a second set of experiments we verified the efficacy of the SINEUP constructs in promoting upregulation of endogenous BDNF protein expression in different mammalian species (rat, human and mouse), in distinct cell types (hippocampal primary cells versus neuroblastoma and astrocytic cell lines) and with complementary techniques to measure SINEUP activity (immunofluorescence and Western blot). First, we transfected rat hippocampal neurons and measured BDNF protein levels by immunofluorescence (Fig. 2A, B Towards this aim, hippocampal neurons were co-trasfected at days in vitro 3 (DIV3) with pEGFP-N1 (Green channel) and SINEUP plasmids (ratio of 1:5). Following cell fixation, immunofluorescent staining against BDNF (red channel) was performed. Images of GFP positive neurons (bona-fide co-transfected with SINEUP plasmid), were acquired with an epifluorescence microscope at 60x magnification in conditions of constant illumination and acquisition time (Fig. 2A). Three regions of interest (ROI) were designed on each GFP positive pyramidal neuron. The first ROI (Figs. 1 and 2B) was drawn on the soma, excluding the nuclei, with a surface dimension ranging from around 40 to 60 square microns. The second ROI (Fig. 2B, n.2) was made by a 2 μm wide line from the apical dendrite hillock to the end of the labelling signal for BDNF. This second ROI was used to perform a densitometric ''profile plot'' of the BDNF staining along the dendrites, considering also the distance from the soma. To measure the background signal intensity, a third ROI (Fig. 2B, n.3) was drawn in a region of each picture devoid of any stained cells. Quantification of anti-BDNF immunofluorescence intensity using an antibody able to recognize both mature and pro-BDNF, subtracted from the background of each image, revealed a highly significant increase in endogenous BDNF expression levels in both somata and dendrites following three days of treatment with either SINEUP-PAN or SINEUP-Ex1 with substantially equivalent effects of the two SINEUP constructs.

To determine the SINEUPs efficiency in enhancing the expression of human BDNF, the plasmids encoding EGFP, SINEUP-PAN or SINEUP-Ex1 were transfected in human SH-SY5Y neuroblastoma cells. This cell line has a basal level of endogenous BDNF [42, 43] and SINEUP-PAN, but not SINEUP-Ex1, was able to significantly increase pro-BDNF 24 h levels (*p* < 0.05; Fig. 2D). The lack of SINEUP-Ex1 activity was expected, due to the absence of Ex1-BDNF transcript expression in this cell line [42].

To monitor SINEUP activity in mouse cells, we used C8-D1A cells, a type I astrocyte clone derived from mouse cerebellum. In addition, we tested SINEUP-PAN2, which shares the same BD as SINEUP-PAN but shortened to 18 nt, spanning positions − 14 to + 4 relative to the M1 AUG. This shorter BD is particularly relevant for the future synthesis of antisense oligonucleotides with SINEUP activity targeting BDNF. Transfection of SINEUP-PAN, SINEUP-PAN2 or SINEUP-Ex1 in mouse C8-D1A cells resulted in an approximately 2.5 fold increase in mature BDNF levels compared to cultures transfected with EGFP alone (Fig. 2E). In conclusion, these results demonstrate that both SINEUP-PAN and SINEUP-Ex1 can promote an increase in endogenous BDNF levels in different mammalian species, distinct cell types irrespective of the different techniques used to measure protein amounts. As expected, only SINEUP-PAN enhances endogenous BDNF expression in the human neuroblastoma cell line SH-SY-5Y which lacks the BDNF transcript containing the exon-1.

### BDNF-specific SINEUPs impact on neuron development and maturation in vitro

**Fig. 3:**
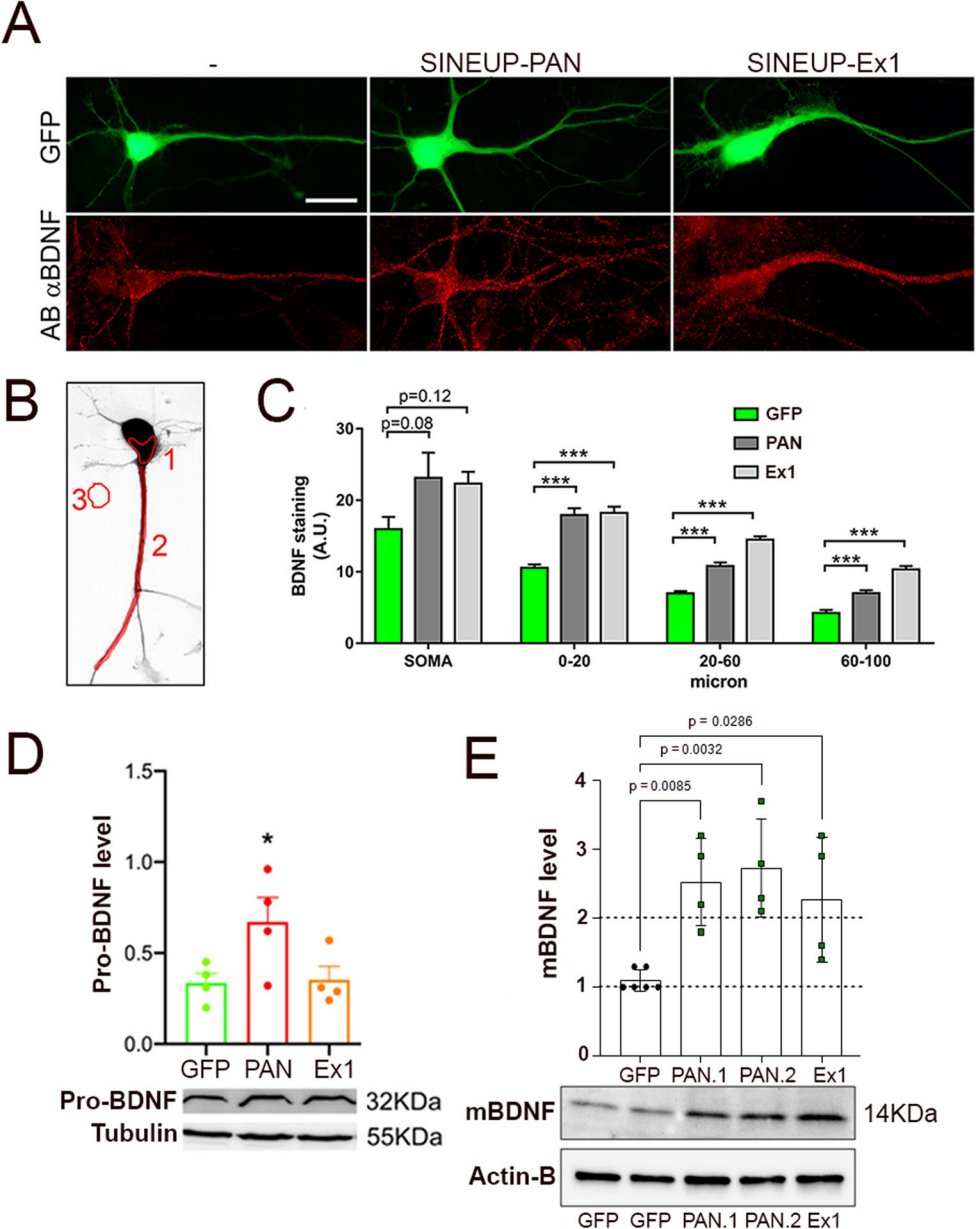
Effects of SINEUPs on rat hippocampal neuron differentiation at different time points in vitro. (**A**) Representative images of DIV12 rat hippocampal neurons in culture after transfection with the control plasmid GFP, SINEUP-PAN or SINEUP-Ex1. Scale bar: 50 μm. (**B**) Average TDL (TDL/NeuN) measured in rat hippocampal cultures at the indicated days in vitro (DIV) in control conditions (GFP) or after transfection at DIV3 with SINEUP-PAN or SINEUP-Ex1 and analyzed at DIV4, 6, 9, 12 as indicated. Data are expressed as % of ratio of TDL on total number of NeuN-positive neurons with respect to control (taken as 100%). *p* = 0.008. (**C**) Average dendrite endpoints measured in rat hippocampal cultures at the indicated days in vitro (DIV) in control conditions (GFP) or after transfection with SINEUP-PAN or SINEUP-Ex1 as indicated. Data are expressed as % of ratio of endpoints on total number of NeuN-positive neurons with respect to control (taken as 100%). *N* = 2, *p* = 0.046

Rat hippocampal neurons in culture show a progressive increase in the total dendritic length (TDL) during the first 6 days in vitro (DIV6), followed by a plateau phase around DIV7-9, and a further progressive increase from DIV10 to DIV15 [44]. To investigate how the development of primary rat hippocampal neurons is affected by the SINEUP-mediated increase in endogenous BDNF, we analysed several morphological parameters at various time points. Rat hippocampal neurons were transfected with SINEUPs at DIV3 and then, to visualize the dendrites, an immunofluorescence analysis was performed at DIV4, DIV6, DIV9, and DIV12 using antibodies specific for the cytoskeleton protein Map2 (Fig. 3A). The number of neurons was quantified with anti-NeuN immunostaining, while the total number of plated cells was obtained by counting nuclei stained with Hoechst-33,342 (Suppl. Figure 3). These data were used to quantify neuronal total dendritic length (TDL) and neurite endpoints, as previously described [45]. When cell morphology was analysed at DIV4, DIV6 and DIV9, no significant increase in the TDL, nor in the number of dendritic endpoints was found in cultures transfected either with SINEUP-PAN or SINEUP-Ex1, with respect to the control (Fig. 3B, C). However, at DIV12, which is the time point at which neurons start to show spontaneous synaptic activity, SINEUP-Ex1 induced a significant increase in TDL (+ 127.5 ± 8.42%; *p* = 0.008), with respect to the control (Fig. 3B), with no changes in neuronal density (expressed as the number of neurons/field) or in the percentage of neurons with respect to the total number of plated cells (Suppl. Figure 2). In addition, cultures at DIV12 transfected with SINEUP-Ex1 showed significantly more endpoints with respect to control cultures (+ 119 ± 8.23%; *p* = 0.046; Fig. 3C). These findings are evidence that treatment of hippocampal neurons with SINEUP-Ex1 from DIV3 promotes an increased complexity of dendritic arborisation at DIV12.

### Effect of SINEUPs in primary rat hippocampal neurons following an Aβ challenge

**Fig. 4:**
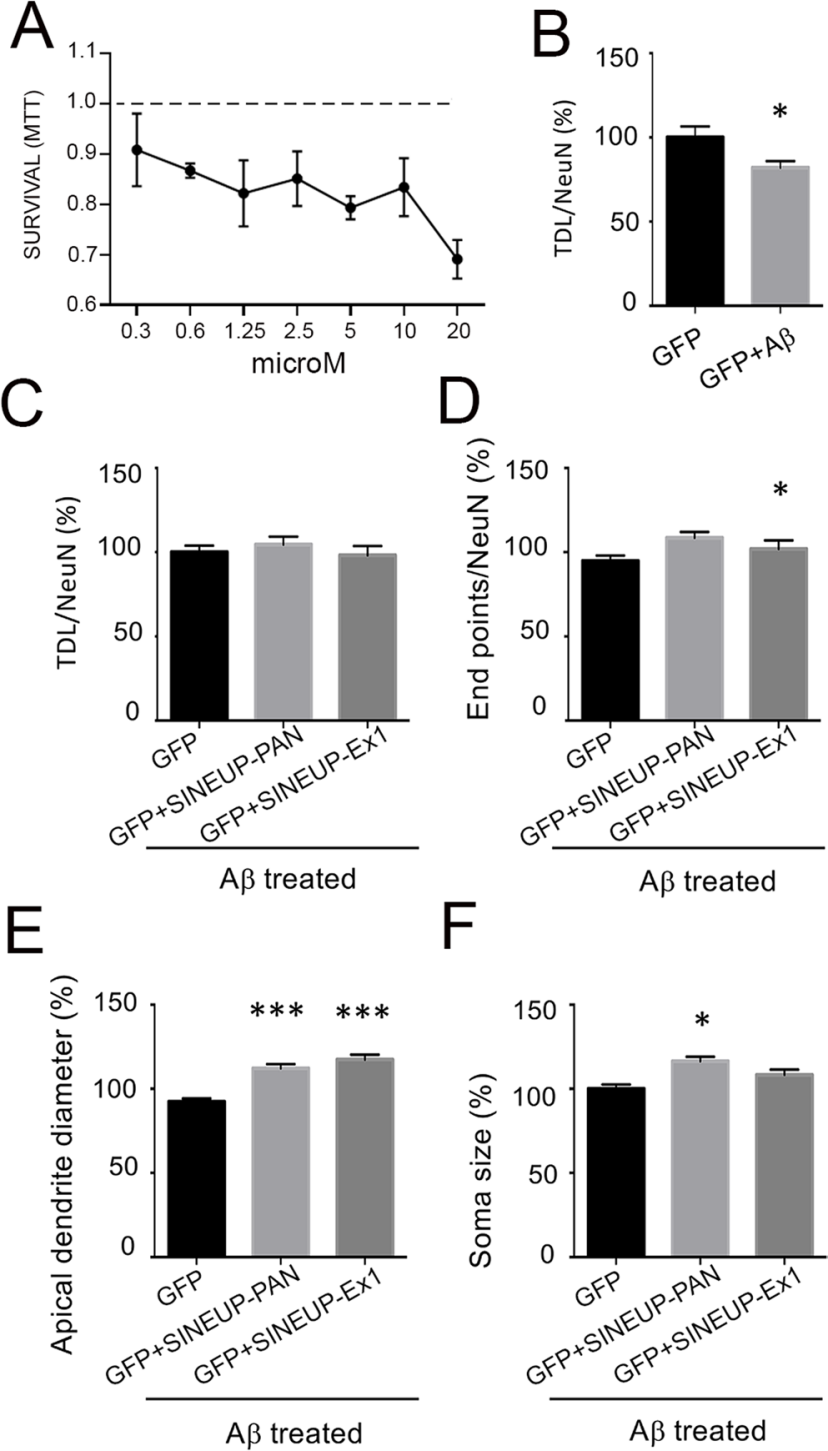
Effect of SINEUPs on Aβ_25−35_ treatment in hippocampal neurons in culture. (**A**) MTT survival assay at increasing concentrations of Aβ_25−35_ for 24 h with respect to control DMSO (dashed line). (**B**) Total dendritic length (TDL) measured on Map2 immunofluorescence signal is significantly reduced after Aβ_25−35_ treatment (10 µM, 24 h) with respect to control-transfected cultures (GFP). *p* = 0.0153. C-F. Effects of SINEUPs on rat hippocampal neurons treated with Aβ_25−35_. Neuronal parameters quantified in neurons treated with Aβ_25−35_ after transfection of an unrelated plasmid (GFP) or after transfection of SINEUP-PAN or SINEUP-Ex1, as indicated. Data are normalised on total number of neurons (NeuN) and are expressed as % with respect to control. *N* = 3 independent experiments, *p* < 0.05, *p* < 0.01,*p* < 0.001, *p* < 0.0001. (**C**) Average total dendritic length (TDL) measured in rat hippocampal cultures in control conditions (GFP) or after transfection with SINEUP-PAN or SINEUP-Ex1 as indicated. (**D**) Dendrite endpoints measured in rat hippocampal cultures in control conditions (GFP) or after transfection with SINEUP-PAN (*p* = 0.0188, ANOVA) or SINEUP-Ex1, as indicated. (**E**) The apical dendrite diameters were evaluated for ~ 100 neurons/condition. Data for SINEUP-PAN (*p* < 0.0001, ANOVA) or SINEUP-Ex1 (*p* < 0.0001, ANOVA) are expressed as average % and normalized to the control condition (GFP without Aβ challenge = 100%). (**F**) The soma sizes were evaluated for ~ 100 neurons/condition. Data for SINEUP-PAN (*p* < 0.0001, ANOVA) or SINEUP-Ex1 are expressed as average % and normalized to the control. Error bars represent SEM

To evaluate the potential neuroprotective effect of BDNF-specific SINEUPs, we induced dendritic atrophy by a challenge with aggregated Aβ_25−35_ oligomers (Aβ) in rat hippocampal neurons in vitro and then we investigated if SINEUP-PAN or SINEUP-Ex1 could rescue the neuronal damage. The Aβ_25−35_ is a peptide of 11 amino acids corresponding to the functional domain of the full length Aβ peptide, and has been demonstrated to have high toxicity in vitro causing neuronal damage [46] and to be present in AD patients brains [47]. Accordingly, we transfected neuronal cultures at DIV3 with SINEUPs or GFP and then we treated them with 10 µM aggregated Aβ_25−35_ oligomers at DIV5 for 24 h. This concentration of Aβ_25−35_ was established with a curve dose-response in MTT assay (Fig. 4A) in order to produce a limited neuronal mortality at 24 h (mortality was around 15–20% NeuN/Hoechst positive cells), in line with the literature in the field [48]. Immunofluorescence staining for Map2 and NeuN was performed at DIV6 to evaluate TDL, neuronal density and dendrite endpoints, apical dendrite diameter and soma size (Suppl. Figure 3). As shown in Fig. 4B, Aβ treatment induced a significant reduction in the average TDL with respect to control, in agreement with previous studies [46]. In particular, after Aβ_25−35_ treatment, the TDL was 82.1 ± 3.901% (*p* = 0.0153) of the control conditions (100 ± 6.573%; *n* = 3; Fig. 4B). While expression of either SINEUPs had no significant effect on TDL (Fig. 4C), the SINEUP-PAN had a significant effect on dendrite endpoints (*p* = 0.0188; Fig. 4D) and on soma size (*p* < 0.0001; Fig. 4F). Moreover, both SINEUP-PAN and SINEUP-Ex1 induced a significant increase (*p* < 0.0001) in the apical dendrite diameter measured at a 20 μm distance from the soma (Fig. 4E).

**Fig. 5:**
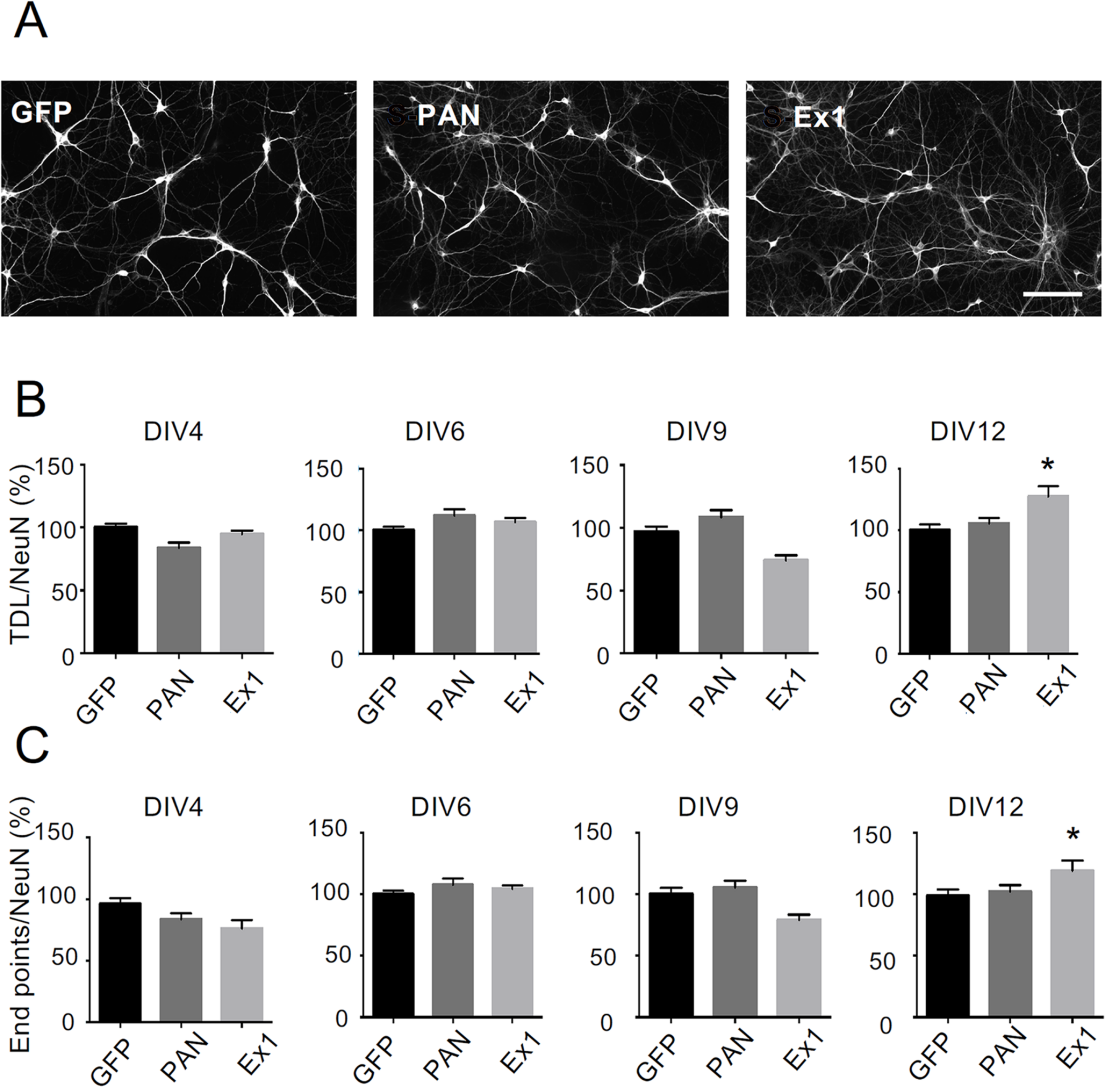
Expression of SINEUPs protects from Aβ-induced cytoskeleton degeneration. (**A**) Representative fluorescence microscopy images of hippocampal neurons in culture, in control conditions (GFP), after incubation with Aβ_25−35_ or transfected with SINEUPs. Map2 signal was used to define the area with the highest fluorescence intensity (area in threshold). The same threshold was applied to all microscopy images. Scale bar: 50 μm. (**B**) Histograms quantify Map2 fluorescence in untreated control neurons (GFP) and in Aβ_25−35_-treated neurons (GFP+Aβ_25−35_). Note degenerated Map2 signal in Aβ_25−35_-treated neurons. *N* = 20 images for condition, *p* < 0.0001. (**C**) Histograms represent Aβ_25−35_-treated neurons in control (GFP) and after SINEUPs transfection. Data are expressed as average % and normalized to the control condition, *p* = 0.0001. **D**) Equal number of neurons per condition was ensured by counting of NeuN positive cells in each image. *N* = 20

Aβ oligomer challenge and mis-sorting of endogenous Tau into the somatodendritic compartment are initial events of AD leading to a depletion of spines and breakdown of microtubules in dendrites [49–51]. Accordingly, we investigated if the recovery by SINEUPs of the atrophic dendrites morphology induced by the Aβ challenge could be explained in terms of microtubules stabilization. Thus, using an automated analysis of Map2 fluorescence based on the definition of a threshold mask applied to each image (see Suppl. Figure 4), we quantified the Map2 intensity per neuron in control and in Aβ treated cultures. We observed a dramatic decrease of the Map2 fluorescent signal, detected according to the defined threshold analysis, in neurons treated with Aβ_25−35_ (56.29 ± 3.0%; *p* = 0.0001) with respect to the control (100 ± 4.9%; Fig. 5B). Significantly, Map2 immunofluorescence intensity was completely rescued in the presence of SINEUP-PAN (148.9 ± 11.58%; *p* = 0.0001), reaching staining levels even beyond control (100 ± 5%; Fig. 5C). This effect is specific because no significant difference in the number of neurons per condition were found (Fig. 5D). Taken together, these data support a positive preventive effect of SINEUP-PAN on microtubule breakdown induced by Aβ_25−35_ treatment in hippocampal neurons.

## Discussion

In this study, we successfully applied the SINEUP technology to enhance intracellular BDNF protein expression and exert a neuroprotective action against an Aβ peptide challenge. To reach this result, we designed two SINEUPs, the first based on sequences complementary to the coding exon, common to all BDNF transcripts (SINEUP-PAN) and the second, complementary to the BDNF 5’ exon-I transcript (SINEUP SINEUP-Ex1). We provide evidence that during development in vitro of primary cultures of rat hippocampal neurons, the SINEUP-Ex1, but not SINEUP-PAN, can induce more complex dendritic arborisation (i.e. greater total dendritic length and higher number of endpoints), an effect which was visible at DIV12, but not at earlier stages. However, following 24 h incubation with aggregated Aβ_25−35_ peptide in DIV6 rat hippocampal neurons, both SINEUP-PAN and SINEUP-Ex1 exerted protection against dendritic atrophy. In fact, SINEUP-PAN promoted the rescue of the number of dendritic endpoints and soma size, and both SINEUP-PAN and SINEUP-Ex1 promoted the rescue of the apical dendrite diameter, while neither of the two SINEUPs had effect on total dendritic length. The protection from the Aβ challenge achieved with the two different SINEUPs seems to involve different mechanisms, as only SINEUP-PAN was able to increase the immunofluorescence for Map2, a marker of dendritic microtubules whose breakdown is part of the pathogenesis of AD. Since SINEUP strategy acts on the translation efficacy of target mRNAs, our results suggest the involvement of specific BDNF transcripts in the rescue from Aβ challenge.

The number of identified long non-coding RNA (lncRNAs) genes have surpassed the number of protein coding ones, increasing the complexity of the mammalian transcriptome. They share many features with mRNAs, as the presence of introns, the epigenetic marks and splice variants. Many lncRNAs overlap coding genes entirely or partially and can originate either from the sense or the antisense strand [52]. Following the discovery of the functions of ncRNAs and of the increasingly complex mechanisms of gene regulation and expression, RNAs are considered as new potential pharmacological agents. The efficacy of the SINEUP in boosting neurotrophic factor levels has been recently proven using this technology to enhance translation of GDNF, demonstrating its ability to rescue motor deficits in a mouse model of Parkinson’s disease [37].

Early intermediate aggregated forms of Aβ are linked to the Alzherimer’s disease pathogenesis [53]. Aβ can assembly in different forms which can be divided in 3 major groups: monomers, soluble oligomers and insoluble fibrils [54]. Different small Aβ fragments, such as Aβ_1−42_ or Aβ_25−35_, show a comparable neurotoxic effect in vitro [55]. In particular, the 11-amino acid-long Aβ_25−35_ peptide used in this study, whose sequence corresponds to the functional domain of the full-length Aβ peptide, contains a hydrophilic domain (Aβ_25−28_) involved in the formation of β-sheet structures, and a hydrophobic domain (Aβ_29−35_) [39]. In vivo studies verified the presence of Aβ_25−35_ in neurons of the subiculum and entorhinal cortex of AD brains [47]. Taken together, these studies indicate that the Aβ_25−35_ peptide is a convenient tool to induce an Aβ peptide challenge in cultured neurons.

Our first set of experiments was focused on verifying if the two SINEUP constructs could enhance translation of BDNF mRNA transcripts in HEK293T cells. We found a significant increase of BDNF protein expression starting from 1:3 ratio of SINEUP versus an unrelated control plasmid coding for the GFP protein. We observed a 4-fold increase of Ex1-BDNF protein expression induced by the SINEUP-PAN and a 5-fold increase induced by the exon I-specific SINEUP-Ex1. Moreover, the SINEUP-PAN showed an upregulation effect on both exon IIB- and exon IIC-BDNF protein expression (3-fold increase and 2-fold increase, respectively).

We then carried out experiments to prove the ability of SINEUPs to increase endogenous BDNF protein levels in different mammalian species, and cell types and according to distinct methods to measure the amount of target protein. First, we tested the SINEUPs on endogenous BDNF expressed in primary rat hippocampal neurons. An intrinsic limitation of our strategy is that the SINEUP constructs do not have a reporter gene and therefore, we were not able to identify how many and which neurons were effectively transfected. When the transfection efficiency was evaluated through pEGFP transfection and counting the EGFP-positive neurons on the total number of cells (Hoechst stained), the transfection efficiency was of ~ 14%, not far to the 20–30% indicated in literature for primary rat hippocampal neurons [56]. These results suggest that the use of a viral delivery system could in principle significantly increase the amount of cells expressing the SINEUP RNA, enhancing SINEUP actvity on endogenous BDNF expression and its biological effects, as previously shown for SINEUP-GDNF [37]. We then found that the SINEUP-PAN, but not the exon I-specific SINEUP-Ex1, can promote a significant increase in endogenous BDNF levels in the human neuroblastoma cell line SH-SY5Y. As we have previously shown [42], in this cell line the BDNF transcript containing the exon 1 is not expressed, thus explaining why SINEUP-Ex1 was unable to boost BDNF expression levels, and providing an excellent control for the specificity of these tools. Furthermore, we tested the activity of SINEUPs in a mouse astrocytic cell lines, demonstrating that all RNAs were able to increase endogenous BDNF expression by approximately 2.5 fold. In the latter experiments, we also evaluated SINEUP-PAN2, a variant of SINEUP-PAN with shortened BD. This study is instrumental for the future design of short (less than 50 nt), chemically sythesized antisense oligonucleotides capable of enhancing BDNF expression to levels comparable to those achieved by plasmid-expressed full-length SINEUPs.

In another set of experiments, we showed that SINEUP-Ex1, but not SINEUP-PAN, was able to increase dendritic arborisation in primary rat hippocampal neurons when transfected at DIV3 and observed at DIV12, with no effect at earlier stages. The result that enhancement of BDNF levels can lead to an increase in dendritic complexity only at DIV12, i.e. at the stage at which synapses start to show spontaneous synaptic activity [44] is in perfect agreement with a large body of literature indicating that BDNF requires neuronal actitity to exert its functions [57]. On the other hand, the lack of effect of the SINEUP-PAN is unexpected because we previously demonstrated that overexpression of a plasmid encoding the BDNF CDS in fusion with GFP was able to promote a robust increase in the number of dendrites after transfection from DIV3 to DIV7 [24]. Instead, results with the SINEUP-Ex1 are in agreement with our own previous data on overexpression of the exon I-CDS which induced higher number of primary dendrites with a 29% increase in the number of crossings at 30 μm from the soma according to the Sholl analysis [24]. A possible explanation regarding the lack of effects in neurons of the SINEUP-PAN, despite its high efficacy in boosting the BDNF levels in HEK293 cells, could be related with intrinsic self-tuning mechanisms, which may prevent translation of BDNF when it reaches too high levels. Indeed, homeostatic regulation of BDNF via miRNA-132 has been described in neurons [58]. However, SINEUP-PAN was found to be efficacious in our second set of experiments, in which neurons were stressed with an Aβ challenge. It is well known that under these stressing conditions, the proteolytic pathways are upregulated and protein biosynthesis is altered [59–62], and therefore it is conceivable that the SINEUP-PAN could enhance BDNF protein levels within a more physiological range.

When we evaluated dendritic integrity in Aβ_25−35_ treated rat hippocampal neurons, we observed a significant impairment on the average neurite length in Aβ_25−35_ -treated neurons, in accordance with previous studies [46]. Specifically, we observed a significant increase of the number of endpoints and soma size following SINEUP-PAN treatment and an increase of apical dendrites diameter following both SINEUP-Ex1 and SINEUP-PAN treatment, while we did not find any significant effect of the two SINEUPs on total dendritic length. Given the positive effect on TDL observed with SINEUP-Ex1 in naïve primary neurons at DIV12 but not at earlier stages, one possible explanation is that the association between BDNF increase and synaptic activity is required to induce elongation of the dendrites. On the other hand, the significant effects detected in the whole population strongly suggest that the trophic effect of the enhanced intracellular expression of BDNF is spread across a large population of neurons in the culture, through enhanced availability of secreted BDNF. Importantly, our results are largely in agreement with previous studies showing that BDNF increase the soma size at DIV3 primary hippocampal neurons in vitro (Ogata et al., 2015) and dendrite complexity at DIV7 following overexpression of individual BDNF transcripts from DIV3 [24]. Taken together, our findings provide convincing evidence that expression of SINEUPs induced protective trophic signalling against Aβ insults.

Our strategy to enhance translation of endogenous BDNF mRNAs is potentially able to revolutionize the gene-therapy approaches to target BDNF in neurodegenerative diseases, especially considering the complexity of the BDNF gene. Indeed, the BDNF gene is transcribed from multiple promoters located upstream to distinct 5’ non coding exons that induce the expression of a heterogeneous population of BDNF mRNA transcripts, proven by the presence of a transcription start site in all BDNF exons [22]. Rat and mouse BDNF gene contain eight 5’ noncoding exons followed by a single 3’ protein coding exon. The functional significance of the multiple BDNF isoforms, that finally produce the same protein, has been linked to their differential subcellular localization and local protein synthesis according to the so called “spatial code hypothesis of BDNF splice variants” [63], based on the observation that exons I and IV remain restricted to the soma and proximal dendrites while exons II and VI can be targeted to distal dendrites [38, 64–68]. Various transgenic mouse models of AD were tested for BDNF expression and most showed significant downregulation of total BDNF mRNA and protein which generally correspond to the findings in post-mortem AD brains [6]. Transcript-specific BDNF alterations in AD have been widely described in literature. For instance, in AD post-mortem brains a significant decrease in BDNF transcripts bearing exon I, II, and IV has been detected in parietal cortex [9]. Given the distinct functions and anatomical distribution of the different BDNF transcripts, and given the selective downregulation of individual BDNF variants observed in AD patients and animal models, we believe that there is a strong case for a targeted therapy to upregulate translation of BDNF from single transcripts. In particular, the BDNF transcript containing the exon I is an interesting target for a gene therapy because it is not only downregulated in AD but is also specifically expressed in the brain and is virtually absent from non-neural tissues [22, 23, 69]. Exon I makes exception with respect to the other BDNF 5’exons because it contains one upstream start codon in frame with the start codon in exon IX thus producing a longer signal peptide, while all other 5’exons code only for untranslated regions and thus using exclusively the canonical exon IX start codon [22, 69]. Furthermore, under basal conditions, exon I has a weak translatability because it is the longest BDNF 5’UTR variant with a length of 640 nt, it has the highest free energy (­222 cal/mol) and contains six uAUGs which can act as competing initiation sites and slow down translation [25]. However, upon stimulation or activation through a specific SINEUP as shown in this study, the use of exon I-specific AUG results in high translation levels of BDNF protein, at least at the same levels than activation of the common translation start site through the SINEUP-PAN.

Strikingly, even if the transfection rate for neuronal cultures is generally low and only a fraction of MAP2-positive neurons were likely transfected, we could nevertheless detect a highly significant neuroprotective effect of SINEUP-PAN for BDNF in the whole culture. BDNF is a soluble protein and the very local - juxtacrine or even autocrine - effect is not the only one. Rather, upon secretion from overexpressing neurons, a general beneficial effect of BDNF on the neighbouring neurons is to be expected. The implication of this striking result is that even a sparse number of transfected neurons could have a meaningful, yet potentially clinically relevant, effect in the context of a gene therapy targeting BDNF with a SINEUP strategy. Accordingly, in this study we propose for the first time the use in AD of a novel technology, called SINEUP to rescue neuronal atrophy through enhancement of translation of transcript-specific BDNF.

## Supplementary information

Below is the link to the electronic supplementary material.

Supplementary Material 1

## Data Availability

Research data are available upon written request to the corresponding author.
