# Supplementary material for: Long non-coding SINEUP RNA enhancing BDNF translation prevents dendritic atrophy following an Aβ-peptide challenge
Source: Cell Mol Life Sci. 2026 Mar 14;83(1):214. doi: 10.1007/s00018-026-06175-4 (PMC13187084; doi:10.1007/s00018-026-06175-4)
Supplement: Supplementary file 1 — Supplementary Material 1 [file 18_2026_6175_MOESM1_ESM.docx]

***Supplementary File Atzori, et al.***

***Submitted to Cellular and Molecular Life Sciences (CMLS), Topical Collection: “Beyond the Code: The Functional Landscape of Non-Coding RNAs”***

**Long non-coding SINEUP RNA enhancing BDNF translation prevents dendritic atrophy following an Aβ-peptide challenge**

**ATZORI Marta^1§^, FABBRETTI Elsa^1§^, CIRACI^1^ Viviana, ZUCCHELLI Silvia^2,#^, BON Carlotta^2^**, **MIKASINOVIC Sanja^1^, BAJ Gabriele^1^, GUSTINCICH Stefano^2^and TONGIORGI Enrico^1,^***

1. Department of Life Sciences, University of Trieste, 34127 Trieste, Italy.

2. Center for Human Technologies, Non-coding RNAs and RNA-based therapeutics, Italian Institute of Technology (IIT), 16152 Genoa, Italy;

***Corresponding author:** Enrico Tongiorgi

Department of Life Sciences, University of Trieste

Via Licio Giorgieri, 5 (Q building) - 34127 Trieste, Italy

Tel: +39 040 558 8724 - E-mail: [tongi@units.it](mailto:tongi@units.it)

ORCID n. <https://orcid.org/0000-0003-0485-0603>

**§ Equally contributing authors**

**# In memoriam**

**Runnig title:**

SINEUP RNA for BDNF rescues dendritic atrophy in AD


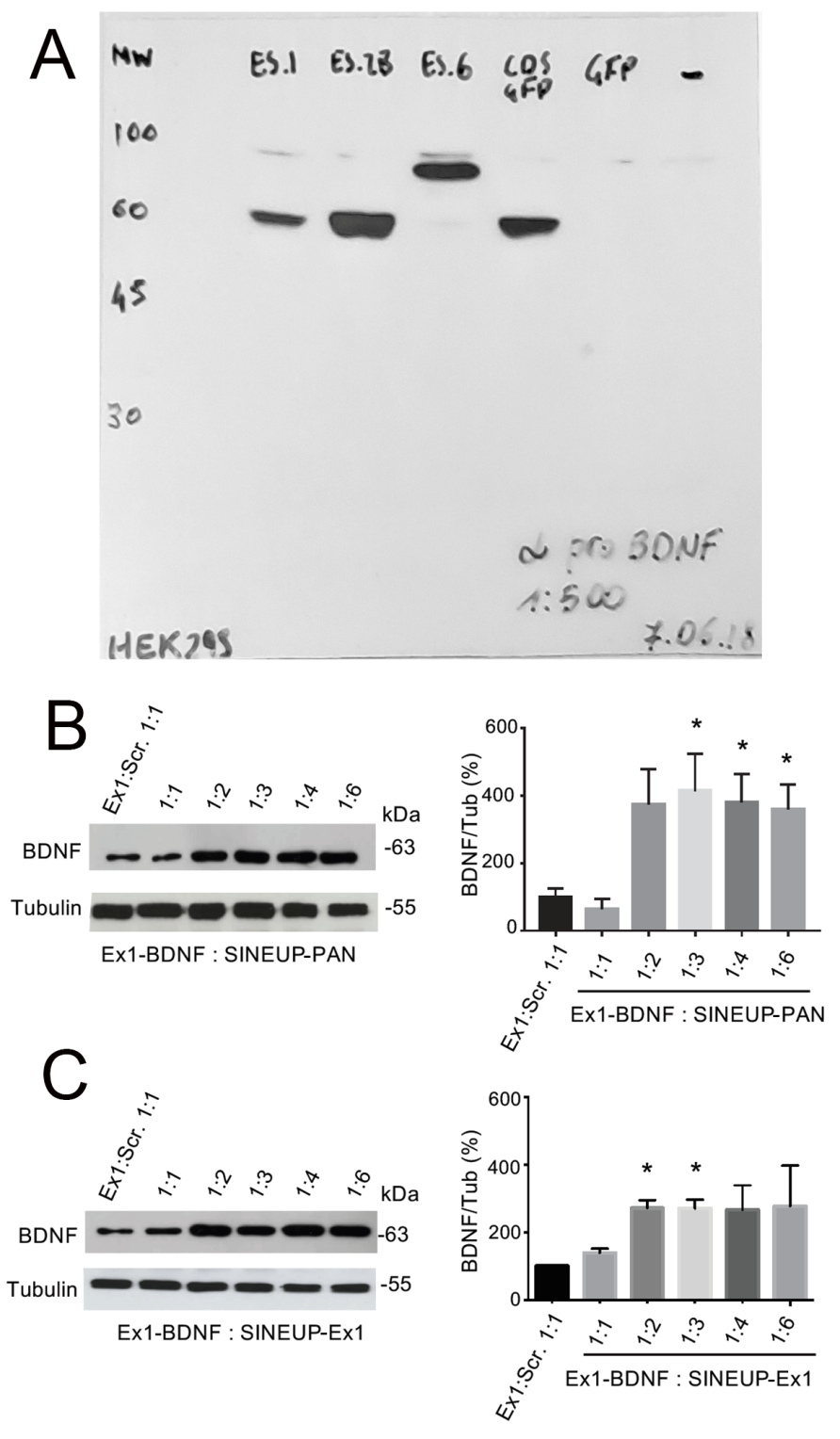


Supplementary Figure 1. **Antibody specificty and analysis of co-trasfection ratio. (A)** Original full picture of the blot with anti-proBDNF (dil. 1:500) on homogenates from HEK293T cells transfected with plasmids expressing either the chimeric Ex1-BDNF-GFP, or Ex2B-BDNF-GFP, or Ex6-BDNF-GFP, or the CDS-BDNF-GFP (with no 3’UTR exon), or GFP alone. The blot clearly shows any signal for BDNF in the cells transfected with GFP alone. Co-transfection ratio of SINEUP-PAN (B) or SINEUP-Ex1 (C) with pEx1-BDNF plasmid in HEK293T cells. Western blot of lysates from extracts of HEK293T cells transfected with Ex1-BDNF with a control plasmid (GFP) or with SINEUP-PAN (A) or SINEUP-Ex1 (B) at the indicated ratio. In the Western blot, BDNF-related signal (expected protein size ~54 kDa) shows expression of BDNF-GFP fusion protein. Tubulin bands (55 kDa) are indicative of gel loading and are used for normalization in the Western blot analys. Histograms quantify average data from n=3 independent experiments. *p<0.05.


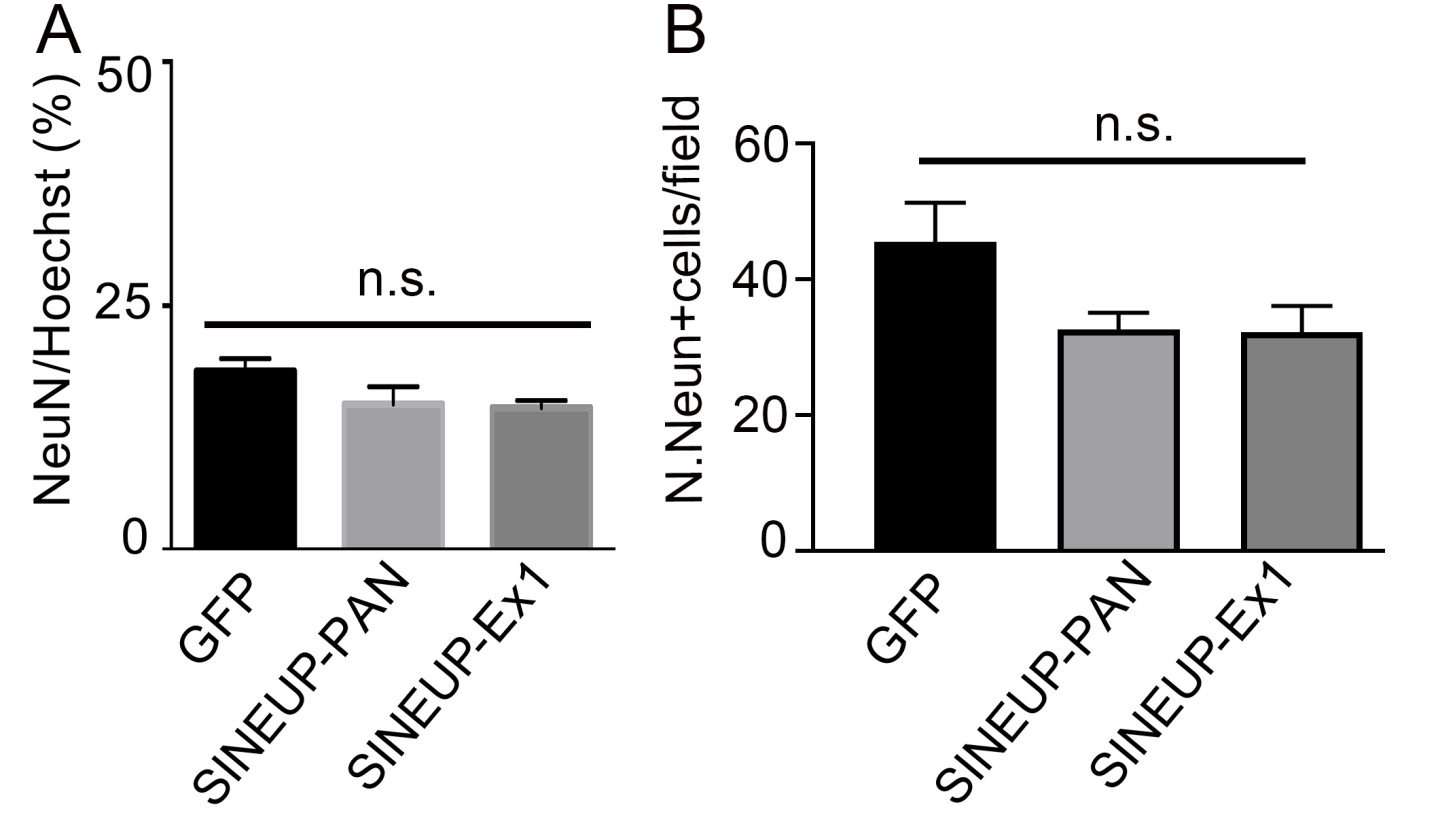


**Supplementary Figure 2. Effects of SINEUP on the percentage and row number of NeuN^+^ neurons at DIV12, transfected at DIV3.** A) Percentage of NeuN^+^ cells (i.e. bona fide neurons) over the total number of cells, identified by the Hoechst nuclear staining. B) Number of NeuN^+^ neurons per field, taken with a 20x objective. N=9 or 12 fields from n=3 different cell cultures. The different groups are not statistically different (one-way ANOVA, with Bonferroni post-hoc comparison between groups).

**
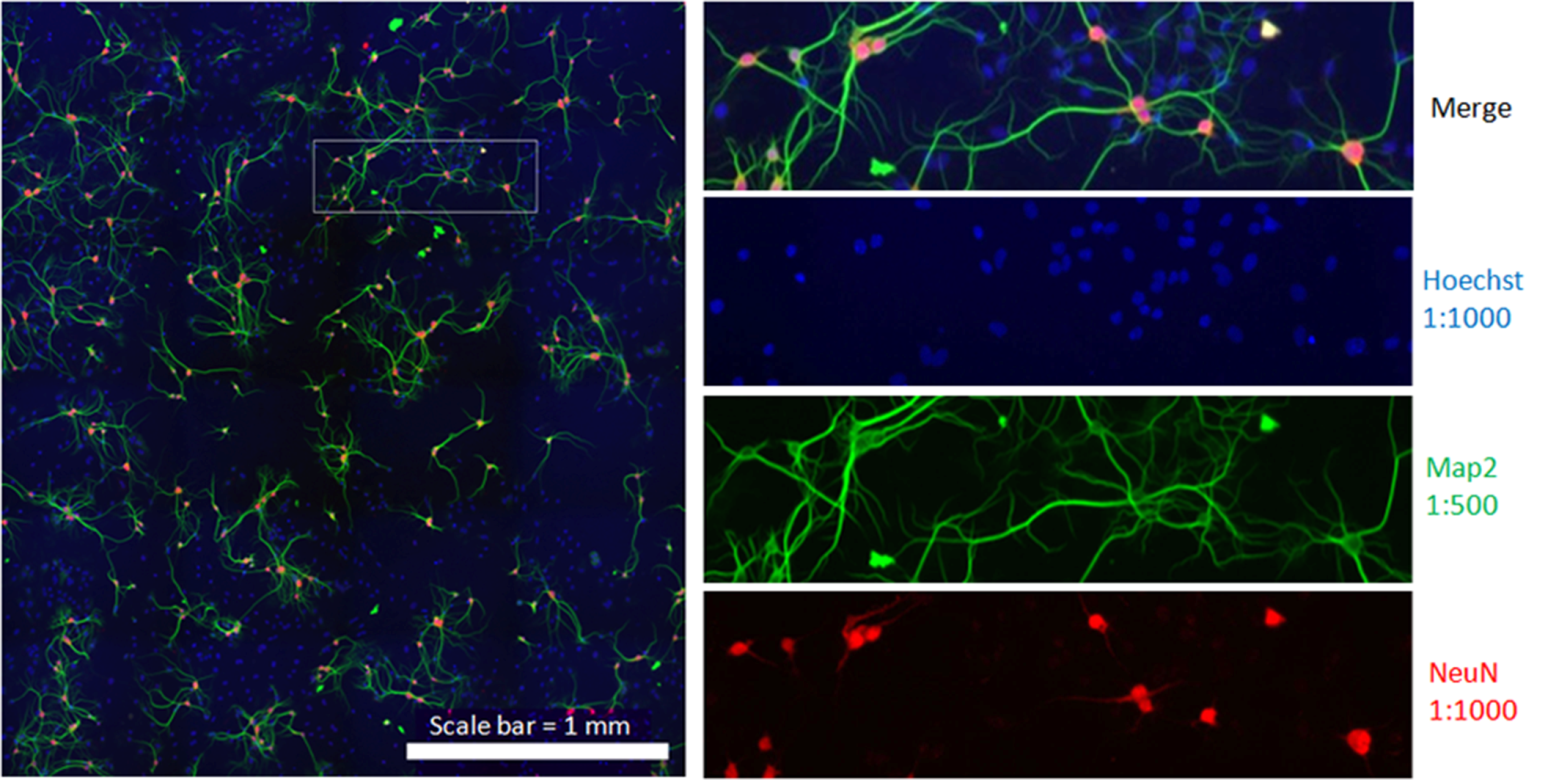
**

**Supplementary Figure 3. Hippocampal neurons at DIV6 staining for NeuriteQuant analysis.** The figure illustrates the staining used to measure the total dendritic length (TDL), the dendritic endpoints, the number of somas and the soma size using the software NeuriteQuant. In the large field on the left, the central region of the culture is shown (4stitched images). The insets at the right, show the staining with Hoechst, Map2, NeuN and the merged image.

**
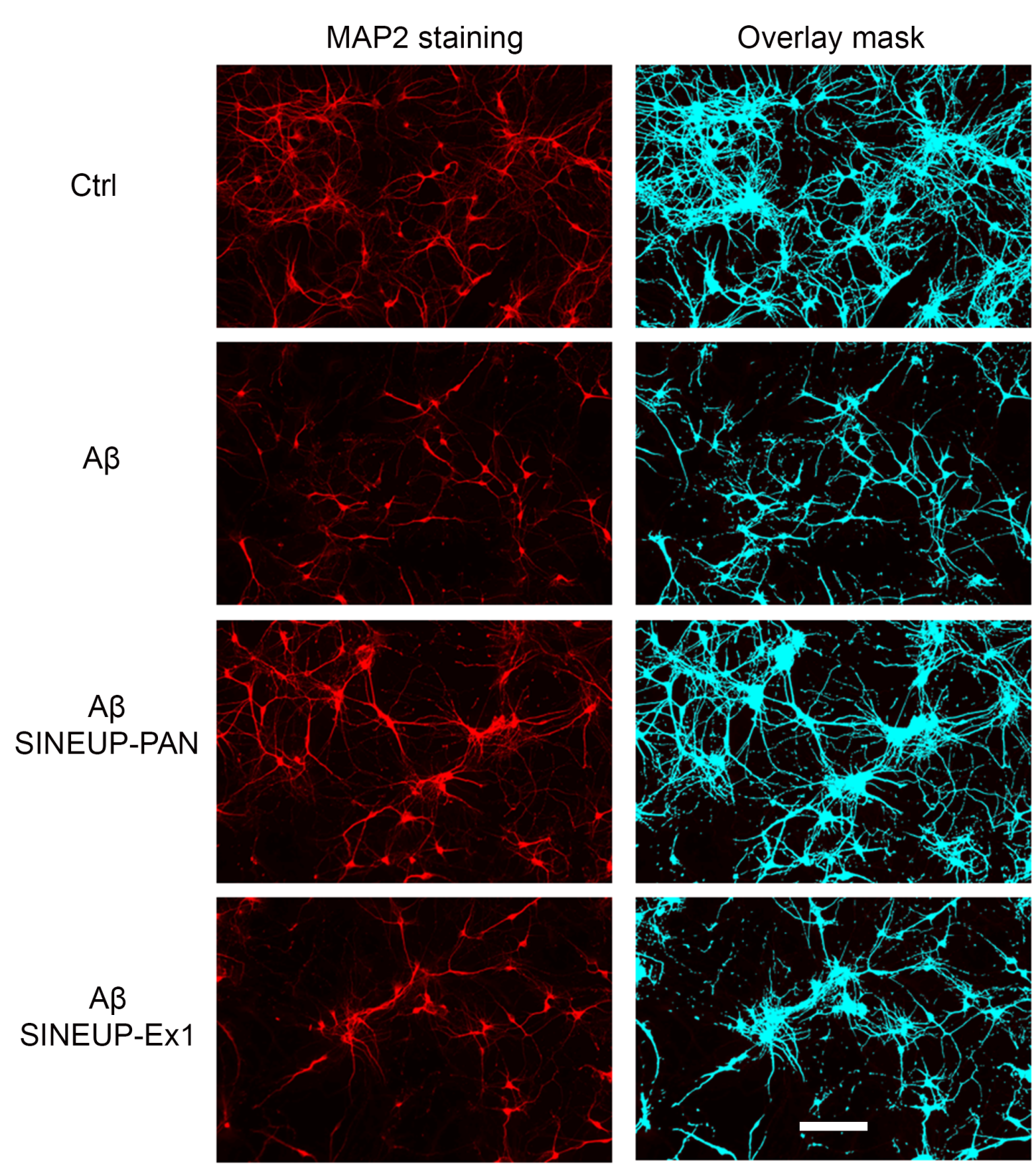
**

**Supplementary Figure 4. Representative fluorescence microscopy images (20X) of untreated neurons, Aβ_25-35_-treated neurons and miniSINEUPs transfected neurons in culture.** Map2 staining (left) was used to define the area with the most fluorescence intensity (right) applied to 20 images for condition
